# Calculated parenteral initial therapy of bacterial infections: Antibiotic treatment in the elderly

**DOI:** 10.3205/id000049

**Published:** 2020-03-26

**Authors:** Peter Walger, Hans Jürgen Heppner

**Affiliations:** 1 Hygiene, Infektionsmanagement und ABS, Bonn, Germany; 2 Johanniter GmbH Berlin, Germany; 3 Verbund Katholischer Kliniken Düsseldorf, Germany; 4 Geriatrische Klinik und Tagesklinik, Helios Klinikum Schwelm, Germany

## Abstract

This is the fifteenth chapter of the guideline “Calculated initial parenteral treatment of bacterial infections in adults – update 2018” in the 2^nd^ updated version. The German guideline by the Paul-Ehrlich-Gesellschaft für Chemotherapie e.V. (PEG) has been translated to address an international audience.

Multimorbidity, an atypical symptomatology of infections in combination with multimedication, the associated interaction risks and serious consequences of synergistic side effects characterize the conditions when deciding on the use of antibiotics in old age. Strict decision regarding the indication itself, choice of the best antibiotic even considering rare side effects which may be dangerous in the elderly, determining the correct dose, dosing interval and the shortest possible duration according to the physiological status of the patient as well as monitoring effectiveness and toxicity detect expected and unexpected side effects early. Recommendations must reflect the peculiarities of antibiotic treatment in elderly patients.

## Zusammenfassung

Dies ist das fünfzehnte Kapitel der von der Paul-Ehrlich-Gesellschaft für Chemotherapie e.V. (PEG) herausgegebenen S2k Leitlinie „Kalkulierte parenterale Initialtherapie bakterieller Erkrankungen bei Erwachsenen – Update 2018“ in der 2. aktualisierten Fassung.

Multimorbidität, eine atypische Symptomatik von Infektionen in Kombination mit Multimedikation, die damit verbundenen Interaktionsrisiken und die oft schwerwiegenden Folgen synergistischer Nebenwirkungen kennzeichnen die Bedingungen bei der Entscheidung über den Einsatz von Antibiotika im Alter. Strenge Indikationsentscheidung, Auswahl des besten Antibiotikums auch unter Berücksichtigung seltener, bei älteren Menschen gefährlicher Nebenwirkungen, Ermittlung der richtigen Dosis, des Dosierungsintervalls und der kürzestmöglichen Dauer entsprechend dem physiologischen Status des Patienten sowie Überwachung von Effektivität und Toxizität erkennen frühzeitig erwartete und unerwartete Nebenwirkungen. Empfehlungen müssen die Besonderheiten der Antibiotika-Behandlung bei älteren Patienten widerspiegeln.

## Introduction

The aging population means that the composition is shifting more and more towards older people. By 2050, it is estimated the proportion of over-65s will be at 30–40%, while at the same time the share of very elderly people (80 years and older) will rise to 15%, almost trebling [1]. This demographic change is leading to an increase in geriatric patients in all areas of medical care. So it is very important to know the basic definition of what constitutes a geriatric patient (see Table 1 (Tab. 1)).

The proportion of 90-year-old patients requiring acute care is also increasing, for example in cardiac surgery [2] or geriatric trauma [3]. This requires a high level of geriatric expertise in care if it is necessary to treat concomitant or complicating infections in this vulnerable patient group [4]. Under adequate treatment the outcome is good while maintaining the patient’s functional level [5]. It is therefore important to have a basic understanding of the peculiarities of anti-infective treatment in the elderly.

Multimorbidity, i.e. the simultaneous presence of several chronic diseases in need of treatment, consequently leads to multimedication and thus to the risk of synergistic side effects and undesirable interactions in antibiotic treatment.

In addition to the physiological changes in the aging human body, immune senescence also leads to a greater susceptibility to infection in this patient group. Morbidity and mortality of many infectious diseases increase with age. Infections are a major cause of death in older patients [6]. 

The most common cause of infection-related death in patients >65 years of age is community-acquired pneumonia (CAP), with long-term care and in particular being bed-ridden significantly increasing the risk of death [7]. Numerous other infections such as urinary tract infections, sepsis, skin and soft tissue infections, bacterial endocarditis, cholecystitis and diverticulitis show increasing incidences. Atypical clinical manifestations, for example by weakening of the fever reaction, nonspecific general symptoms or early impairments of brain function make the diagnosis more difficult and delay timely and adequate treatment [8], [9], [10].

Older patients with bacterial infections have different clinical characteristics compared to younger patients due to their limited local and systemic responses to an infection. Symptoms of acute infection in elderly patients are usually “nonspecific” and “atypical”. Fever, the cardinal symptom of a bacterial infection, is absent in over 30% of cases [11]. The usual lab markers for the detection of an infection, such as leukocyte count or C-reactive protein (CRP), may initially also be normal or only minimally altered [12]. CRP and white blood cell counts are therefore unreliable parameters in geriatric patients [13].

## Key on antibiotic-associated side effects in old age

Physiological changes affect pharmacokinetics in old age. This concerns both the release and absorption of drugs as well as their distribution through the changes to fat and water mass in old age and the (mainly renal) drug elimination. A summary of the most important age-related changes affecting the pharmacokinetics of anti-infective agents is shown in Table 2 (Tab. 2).

Over 30% of people >70 years old have at least five chronic diseases [10]. Extensive use of prescribed and over-the-counter (OTC) medication and herbal preparations is typical of the elderly. For example, 25% of women over the age of 65 (in the US) take five prescribed medications and 12% use ten or more medications. This inevitably increases the risk of complications [14].

The data for Germany are similar. People over the age of 70 take an average of three different medicines per day; those aged 80 to 85 receive the highest number per day [15], 35% of those >70-year-olds receive 5 to 8 and 15% more than 13 different drugs [16]. Of the elderly studied, 14% (in 1998) also took additional herbal or other dietary supplements [17]; in 2002 this figure was 26–27% [18], [19].

A fictitious 79-year-old patient with five of the most common comorbidities (COPD, type 2 diabetes mellitus, hypertension, osteoporosis, osteoarthritis) receives 12 drugs a day according to guidelines (USA 2005), following complicated rules for administration with unpredictable interactions between diseases and medications and with numerous adverse drug reactions [20].

Side effects of medications are generally up to 3 times more common in elderly patients compared to 30 year olds [21]. When taking up to 5 medications, there is a 4% risk of ADR (adverse drug effects), with 6–10 medications this risk is 10% and with 11–15 medications 28% [22]. 

 Overall, ADRs are found in 14.6–35% of elderly patients. 20–25% of geriatric in-patient admissions are the result of ADRs. Anticoagulants, nonsteroidal anti-inflammatory drugs (NSAIDs), antidiabetics, diuretics and digitalis glycosides are most commonly associated with ADRs that lead to hospital admission [23]. Interactions play a role in around 40% of ADRs. Low body weight is associated with ADRs especially often. Over 80% of ADR-associated in-patient admissions are preventable [24], [25].

## Prevalence of inadequate regulations

Data from the US, Canada and Europe show a high proportion of “potentially inadequate medications” (PIM) in elderly patients, for example in the USA 1994: 23.5% USA 1996 20% – of which 3% from the Beers list, historically the first published PIM list – of the 11 “always avoid” drugs with increased risk of hospitalization and death), USA 2002: 19% Europe 2005: ~20%. The typical polypharmacy patient with an increased incidence of inadequate medication is female, >85 years old, living alone and has low health and social status [26], [27].

The Beers Criteria [28] include a list of inadequate medications consisting of three groups: “always avoid” (11 drugs), “rarely appropriate” (8 drugs) and “some indication but often misused” (14 drugs). Based on these criteria, revised PIM lists have been published in the US, France, the Netherlands and Canada [29], [30], [31], [32]. The risk of ADR leading to in-patient admission of elderly patients is therefore particularly high if several medicines are taken at the same time (risk of interaction). Multimedication, prescription of neuroleptics or anti-dementia drugs are significant risk factors for ADRs in nursing home residents [33]. The PRISCUS list has been tailored to the conditions in Germany. It comprises 83 drugs from 18 different drug classes, hailing from a wide range of treatment areas, that are considered potentially unsuitable for senior citizens [34], [35]. However, there is no definite position on antibiotics with the exception of nitrofurantoin.

Polypharmacy generally increases the risk of adverse events and increases the risk of clinically relevant drug interactions. This is also described for some anti-infective drugs which are added to existing multi-medication. To minimize these potential risks, this issue should receive increased attention in making decisions about anti-infective drug treatment.

The treatment of elderly patients with a bacterial infectious disease therefore typically means adding a further substance, an antibiotic, to a long list of different medications with a partially unclear potential for interactions and various ADRs, which may themselves have their own side effects as well as their own potential for interactions.

This can then quickly lead to situations requiring treatment and that are sometimes life-threatening [36].

## Antibiotic prescriptions for elderly patients

The assessment of the different classes of antibiotics (or individual substances thereof) is based on specific aspects and risks adapted to the age of the patient (see Table 3 (Tab. 3)).

## Assessment of renal function in old age

Changes to renal elimination represent the most clinically significant change in body function in terms of pharmacokinetic effects on drugs and is inevitably associated with increasing age [37]. The average renal blood flow decreases by about 10% per decade of age from 600 ml/min per 1.73 m^2^ in the 4th decade to about 300 ml/min per 1.73 m^2^ in the 9^th^ decade. At the same time, the glomerular filtration rate decreases by about 10% per decade. At the same time, as creatinine production decreases with age as a result of the progressive loss of muscle mass, the serum creatinine level remains constant. Creatinine levels in the upper normal range therefore indicate an already existing restriction of kidney function. An increase in serum creatinine should be given special consideration when determining antibiotic dosages. Many labs report the Glomerular Filtration Rate (GFR) based on calculation using the MDRD (Modification of Diet in Renal Disease Study) formula. However, this formula was not validated in the MDRD study regarding people over the age of 70. The alternative formula according to Cockcroft-Gault shows significant limitations of the GFR calculation depending on the age and large variances of the body weight. In comparative studies, GFR estimates based on a 24-hour composite urine sample showed the best results, albeit with a tendency to overestimate. In practice, however, there are significant limitations of the method due to collection errors. Another alternative is the determination of cystatin C, which is characterized by independence from age and muscle mass [38], although discussion of this parameter is also controversial [39]. This method seems to be most reliable in cases of incipient kidney function restriction where there is still no increase in creatinine. Overall, all methods of determining renal function in the elderly have clear limitations. An overestimation of the glomerular filtration rate should therefore be compensated for by a cautious use of potentially nephrotoxic substances [40].

## Antibiotic resistance in old age

All factors associated with the risk of colonization or infection by multidrug-resistant agents are becoming increasingly important in old age. Multimorbidity and specific comorbidities such as diabetes mellitus or COPD, previous antimicrobial treatment, prior hospital stays, being cared for in nursing homes, rehabilitation facilities and other tertiary care structures, carriers of invasive devices such as enteral feeding tubes, central venous indwelling catheters, tracheostomy and urinary catheters, other out-patient nosocomial risks such as dialysis, chronic ulcers or other long-term care and pre-existing colonization accumulate with increasing age. As expected, residents of nursing homes show an increased incidence of resistant pathogens, depending on their functional limitations [41], [42]. The risk of multidrug resistance in the case of an infection poses special requirements for the prescription of adequate antibiotic treatment, be it through the selection of a suitable broad-spectrum antibiotic or through a suitable combination strategy. With inadequate treatment, there is a risk of prolonged hospitalization, increased costs and, in the worst case, increased hospital mortality [43], [44]. The risk of multidrug resistance has to be assessed individually, a general assumption of an age-related increased risk of MRE without appreciation of the individual risk factors leads to inadequate over-treatment with broad-spectrum antibiotics.

## Summary

In principle, the use of antibiotics in elderly patients can be based on the same principles as for younger people. There is no antibiotic which in principle must be regarded as inadequate for older people.

However, the choice and dosage of antibiotics must be adjusted to the general medical problems of old age in conjunction with the physiological changes. The increased risk of resistant and multi-drug resistant infectious pathogens as a result of multiple hospital stays and prior antibiotic treatment has become an increasingly prominent issue in recent years.

Due to the more frequent and potentially serious consequences of antibiotic side effects (adverse drug reactions, ADRs) in older compared to younger patients, the following steps for optimization are essential: Strict decision regarding the indication itself, choice of the best antibiotic even considering rare side effects which may be dangerous in the elderly, determining the correct dose, dosing interval and the shortest possible duration according to the physiological status of the patient as well as monitoring effectiveness and toxicity detect expected and unexpected side effects early. In geriatric patients their frailty carries a great risk of developing a complicated progression with more difficult convalescence and higher mortality [45]. Therefore, recommendations must reflect the peculiarities of antibiotic treatment in elderly patients.

## Note

This is the fifteenth chapter of the guideline “Calculated initial parenteral treatment of bacterial infections in adults – update 2018” in the 2^nd^ updated version. The German guideline by the Paul-Ehrlich-Gesellschaft für Chemotherapie e.V. (PEG) has been translated to address an international audience.

## Competing interests

The authors declare that they have no competing interests.

## Figures and Tables

**Table 1:**
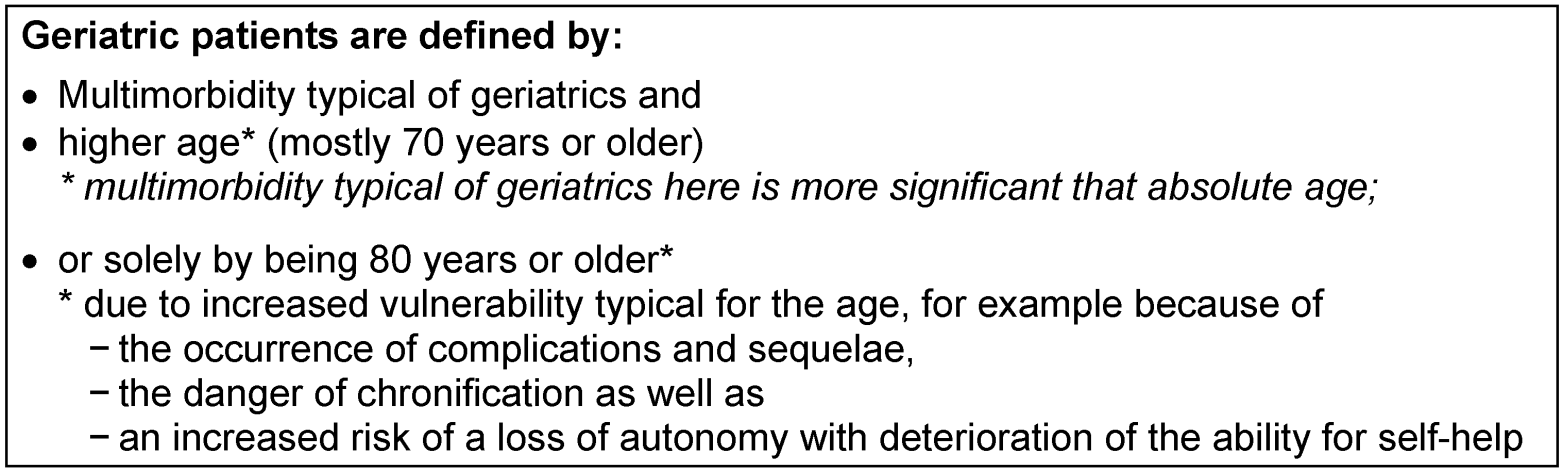
Definition of the geriatric patient [46]

**Table 2:**
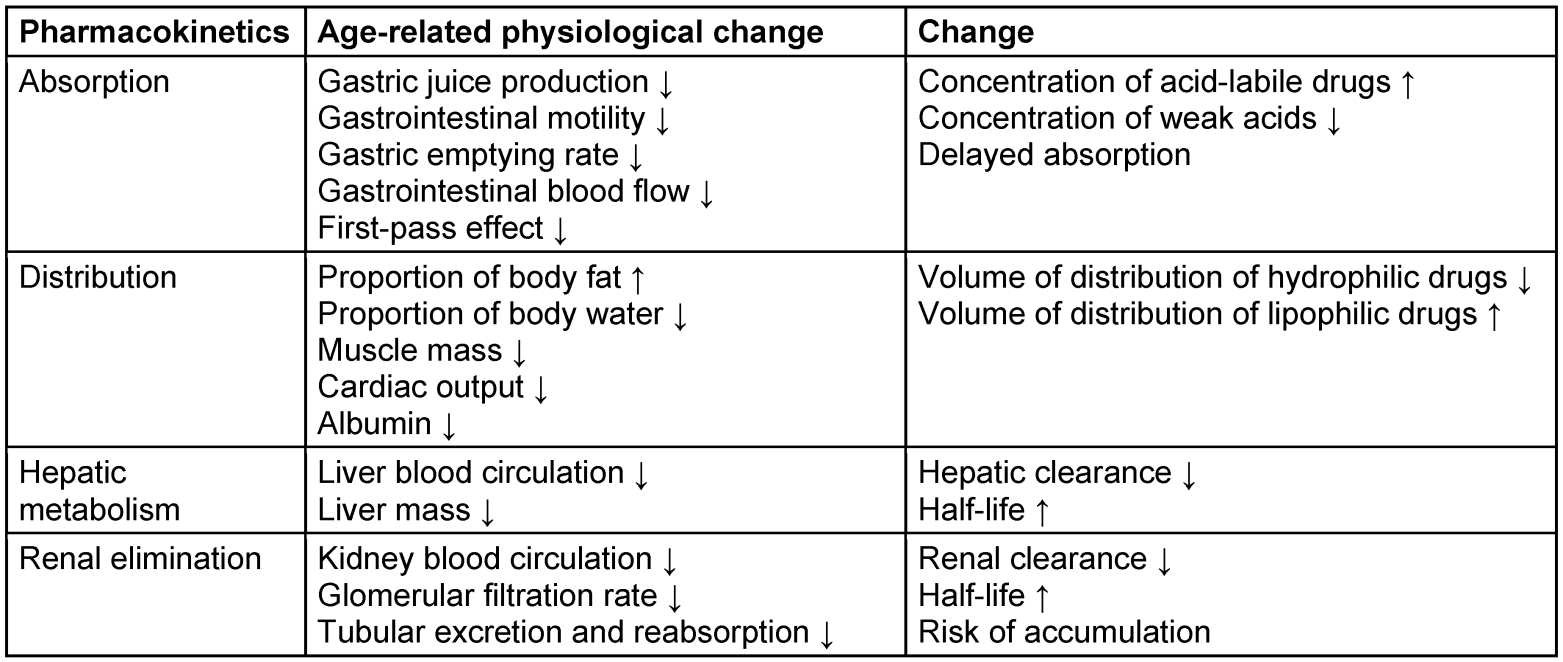
Pharmacokinetics and physiological aging

**Table 3:**
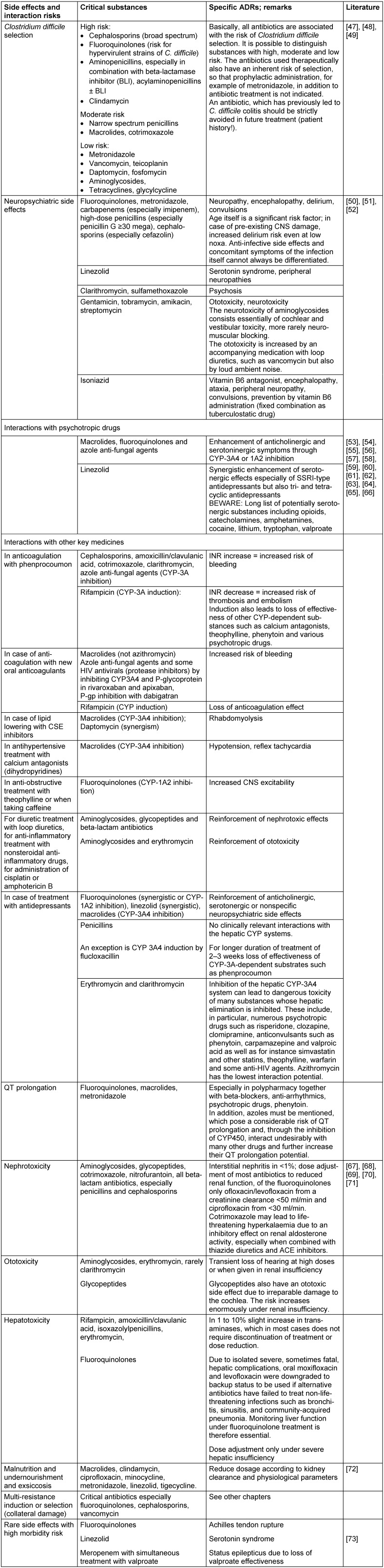
Evaluation of the individual antibiotic classes or individual substances according to side effects and risk of interactions in elderly patients
